# Facilitation of Reparative Dentin Using a Drug Repositioning Approach With 4-Phenylbutric Acid

**DOI:** 10.3389/fphys.2022.885593

**Published:** 2022-05-04

**Authors:** Eui-Seon Lee, Yam Prasad Aryal, Tae-Young Kim, Ji-Youn Kim, Hitoshi Yamamoto, Chang-Hyeon An, Seo-Young An, Youngkyun Lee, Wern-Joo Sohn, Jae-Kwang Jung, Jung-Hong Ha, Jae-Young Kim

**Affiliations:** ^1^ Department of Biochemistry, School of Dentistry, IHBR, Kyungpook National University, Daegu, Korea; ^2^ Department of Dental Hygiene, Gachon University, Incheon, South Korea; ^3^ Department of Histology and Developmental Biology, Tokyo Dental College, Tokyo, Japan; ^4^ Department of Oral and Maxillofacial Radiology, School of Dentistry, IHBR, Kyungpook National University, Daegu, Korea; ^5^ Pre-Major of Cosmetics and Pharmaceutics, Daegu Haany University, Gyeongsan, South Korea; ^6^ Department of Oral Medicine, School of Dentistry, IHBR, Kyungpook National University, Daegu, Korea; ^7^ Department of Conservative Dentistry, School of Dentistry, IHBR, Kyungpook National University, Daegu, South Korea

**Keywords:** cavity preparation, chemical chaperone, dentin-pulp complex, drug repositioning, reparative dentin formation

## Abstract

For hard tissue formation, cellular mechanisms, involved in protein folding, processing, and secretion play important roles in the endoplasmic reticulum (ER). In pathological and regeneration conditions, ER stress hinders proper formation and secretion of proteins, and tissue regeneration by unfolded protein synthesis. 4-Phenylbutyric acid (4PBA) is a chemical chaperone that alleviates ER stress through modulation in proteins folding and protein trafficking. However, previous studies about 4PBA only focused on the metabolic diseases rather than on hard tissue formation and regeneration. Herein, we evaluated the function of 4PBA in dentin regeneration using an exposed pulp animal model system via a local delivery method as a drug repositioning strategy. Our results showed altered morphological changes and cellular physiology with histology and immunohistochemistry. The 4PBA treatment modulated the inflammation reaction and resolved ER stress in the early stage of pulp exposure. In addition, 4PBA treatment activated blood vessel formation and TGF-β1 expression in the dentin-pulp complex. Micro-computed tomography and histological examinations confirmed the facilitated formation of the dentin bridge in the 4PBA-treated specimens. These results suggest that proper modulation of ER stress would be an important factor for secretion and patterned formation in dentin regeneration.

## Introduction

The endoplasmic reticulum (ER) functions as a site for protein folding, lipid biosynthesis, and calcium homeostasis (Sarvani et al., 2017). Numerous proteins, either destined for cell-surface or for secretion, undergo process of synthesis and modification such as folding and assembly (Schröder and Kaufman, 2005). Trafficking and translocation of proteins in the ER would cause loads of stress to the ER regulation due to the large number of proteins that need to be executed (Hartl and Hayer-Hartl, 2009). However, several molecular chaperones, such as multi-domain families of proteins, are located at the lumen of the ER, which help in achieving correct protein folding and assembly (Saibil, 2013). The process of balancing between the load of protein synthesis and its protein folding ability is referred to as ER homeostasis. When there is a disruption in ER homeostasis, ER stress can be triggered which is caused by the presence of mis-folded and unfolded proteins (Ron and Harding, 2012). The unfolded protein response (UPR) activates in response to ER stress when ER capacity for protein folding is deficient (Kozutsumi et al., 1988; Zimmer et al., 1999). UPR activation alleviates ER stress by increasing ER abundance, decreasing synthesis of proteins, and augmenting the ER chaperone protein synthesis to sustain homeostatic balance; however, when ER stress could not be restored by UPR, apoptosis takes place (Merksamer and Papa, 2010; Hetz, 2012).

Dentin is typically classified into formation stages, namely: primary, secondary, and tertiary (Goldberg et al., 2011). During odontogenesis, primary dentin is formed by odontoblasts until the tooth becomes functional. Secondary dentin is formed after root completion and continues lifelong. Particularly, in the pathological condition, odontoblasts form a specific dentin—the tertiary dentin. Tertiary dentin, whether reactionary or reparative, is formed in response to an external stimulus, caries, or abrasion (Angelova Volponi et al., 2018). Reactionary dentin is formed from pre-existing odontoblast, and reparative dentin, which is elaborated by odontoblast-like cells form after the death of the original odontoblasts (Cajazeira Aguiar and Arana-Chavez, 2007; Goldberg et al., 2011). Proper regeneration of dentin still remains as an unmet solved problems in dental fields. Tissue regeneration requires high fidelity of restoration of structure including vesicle secretion for tissue function (Simon and Smith, 2014; Angelova Volponi et al., 2018). Odontoblasts and pulp cells react against the damage through reinitiating differentiation of odontoblasts from stem cells and progenitor cells in dental pulp resulting secretion of the reparative dentin matrix for normal physiologic function of tooth (Simon and Smith, 2014; Angelova Volponi et al., 2018). During dentin formation, ER stress regulation plays a significant role especially in the secretory stage (Cameron et al., 2015). Previous report suggested that ER stress would affect formation of dentin. For instance: when ER stress regulator gene, Tmbim6 was knocked out, the dentin and enamel structures were disturbed by altering odontoblast and enamel differentiation (Aryal et al., 2019). Mutated dentin forming genes of which could cause Dentinogenesis Imperfecta and Dentin Dysplasia may cause a high level of ER stress (Brookes et al., 2014; Brookes et al., 2017; Liang et al., 2019). Odontoblasts and pulp cells react against the damage by reinitiating differentiation of the odontoblasts from stem cells and progenitor cells in the dental pulp resulting in the secretion of the reparative dentin matrix for normal physiologic function of the tooth (Brookes et al., 2014).

4-Phenylbutyric acid (4PBA) is an FDA-approved drug intended to treat congenital diseases in the urea cycle (Iannitti and Palmieri, 2011). However, 4PBA is a known chemical chaperone that functions as an ER stress antagonist (Zeng et al., 2017); a low-molecular weight compound which stabilizes protein conformations, enhancing the protein folding ability of ER and protein trafficking to alleviate ER stress (Yam et al., 2007). A number of previous studies have also proved the therapeutic effects of 4PBA in proteostasis. Mainly, 4PBA facilitates metabolic syndrome and metabolism such as obesity and diabetes (Ozcan et al., 2009; Luo et al., 2010; Wu et al., 2020). The chemical chaperone 4PBA is also known to alleviate ER stress-mediated cell death and genetic disorders caused by protein mis-folding (Sawada et al., 2008; Singh et al., 2008) and protein mis-folding genetic disorders (Vij et al., 2006; Singh et al., 2008). Furthermore, various effects of 4PBA in inflammatory disorders, neurological diseases, and cancers are also well known (Luo et al., 2010; Kim et al., 2013).

Pulp cavity preparation is the most well-known method of dentin-pulp restoration. In this procedure, the tooth itself receives stress even in protein modulation and secretion of ER. This study hypothesizes that 4PBA, not applied in dental treatment yet, is involved in the modification and secretion of protein matrix for dentin formation by attenuating ER stress when added to the process of pulp cavity preparation.

## Materials and Methods

### Animals

All experiments involving animals were performed in accordance with the guidelines of the Kyungpook National University, School of Dentistry, Intramural Animal Use and Care Committee (KNU-2015-136) as previous study mentioned (Jung et al., 2017). For the pulp access cavity preparation, at least 15 adult 8-week-old male ICR mice in each group were euthanized 3, 5, and 42 days after pulp exposure.

### Histology and Immunohistochemistry

The mice were sacrificed after 3, 5, and 42 days from drug treatment for immunological and histological analyses as described previously (Jung et al., 2017; Aryal et al., 2021). Primary antibodies were directed against NESTIN (1:400; cat. no. ab11306; Abcam), glucose regulatory protein 78 (GRP78; 1:400; cat. no. ab21685; Abcam), HRD1 (1:400; cat. no. NB100-2526; Novus Biologicals), MPO (1:200; cat. no. bs-4943R; Bioss), CD31 (1:100; cat. no. AF3628; R&D System), and TGF-β1 (1:100; cat. no. ab92486; Abcam), and the secondary antibodies used in the present study were biotinylated goat anti-rabbit or anti-mouse immunoglobulin G. Immunocomplexes were visualized using a diaminobenzidine tetrahydrochloride reagent kit (cat.no. C09–12; GBI Labs).

### Pulp Cavity Preparation

The animal experiment was performed as previously described (Jung et al., 2017). The animals were anesthetized by intraperitoneal injections of Avertin (Sigma-Aldrich, USA). Pulpal cavity was mechanically prepared in pulp chamber of first right molar of 8 weeks male mice using a 0.6-mm round burr in a high-speed handpiece with water spray under a dissecting microscopy (S6, Leica). Pluronic F-127 medium containing 100 μM 4PBA (experimental) or 0.07% DMSO (control) were locally delivered using Hamilton syringe to the pulpal cavity. The left upper molars were used as the untreated controls. After treatment, the exposed teeth were double-sealed with Dycal (Dentsply Caulk, Milford, DE) and light-cured composite resin with a bonding system. The drug was treated for 3, 5, and 42 days (Jung et al., 2017; Aryal et al., 2021).

### Micro-CT Imaging

The samples after 6 weeks were analyzed using micro-CT imaging (Skyscan1272; Bruker, Kontich, Belgium). The specimens were scanned through 360^o^ at a spatial resolution of 4904ⅹ3280 pixels with a pixel size of 2 mm. The image data were reconstructed and analyzed using Dataviewer and CTAn (Bruker) to quantify the volume of hard tissue formation (Jung et al., 2017).

## Results

### Altered Histological Structures and Localization Patterns of ER Stress Markers

**FIGURE 1:**
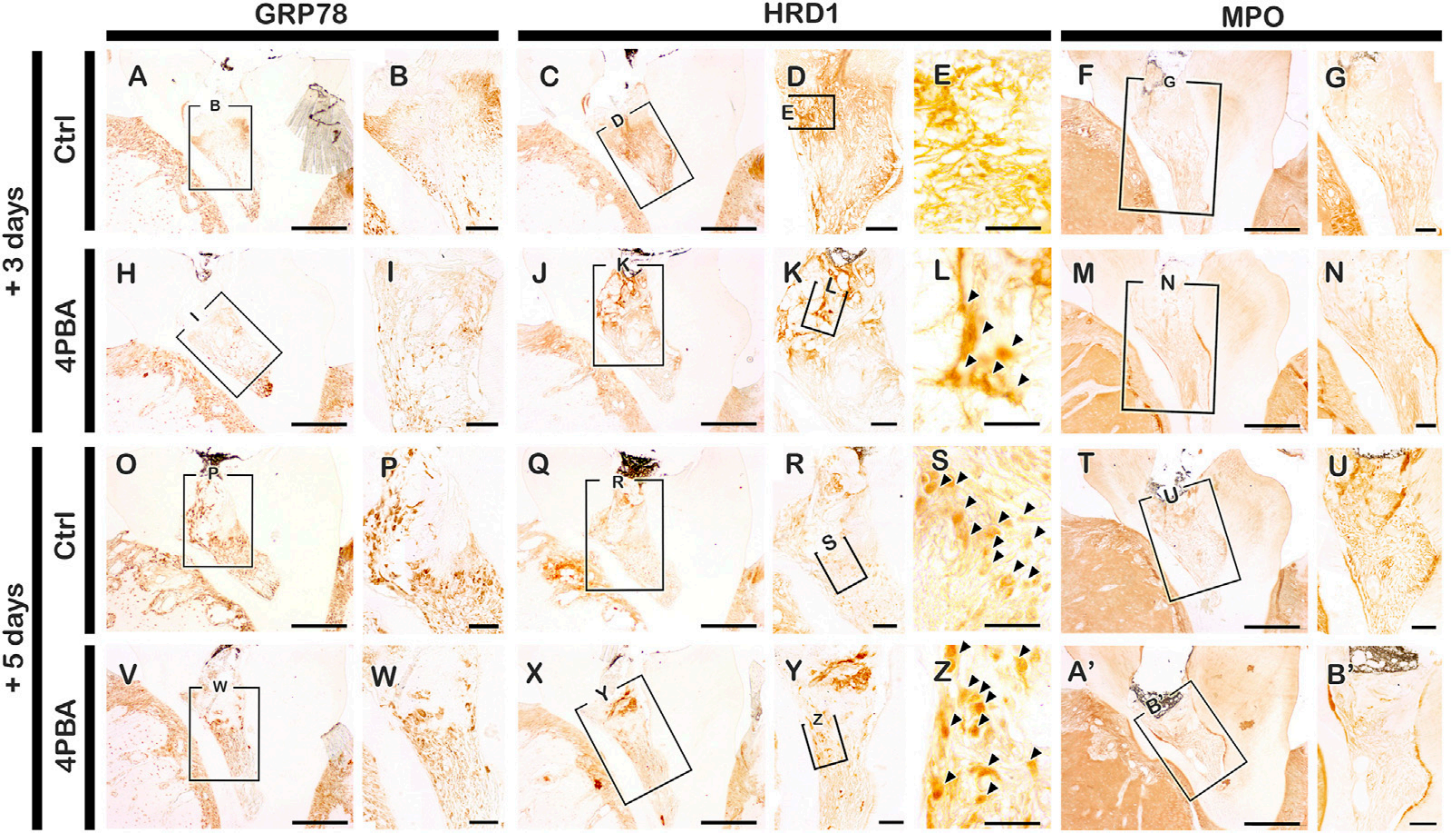
Immunohistochemistry staining with GRP78, HRD1 and MPO after 3 and 5 days pulp access preparation. Arrowhead indicates nucleic localization patterns of HRD1 immunostaining (E, L, S, Z). Boxes in A, C-D, F, H, J-K, M, O, Q-R, T, V, X-Y, A′ indicate enlarged view. Scale bars: A, C, F, H, J, M, O, Q, T, V, X, A’: 200 μm; B, D-E, G, I, K-L, N, P, R-S, U, W, Y-Z, B’: 50 μm.

**FIGURE 2:**
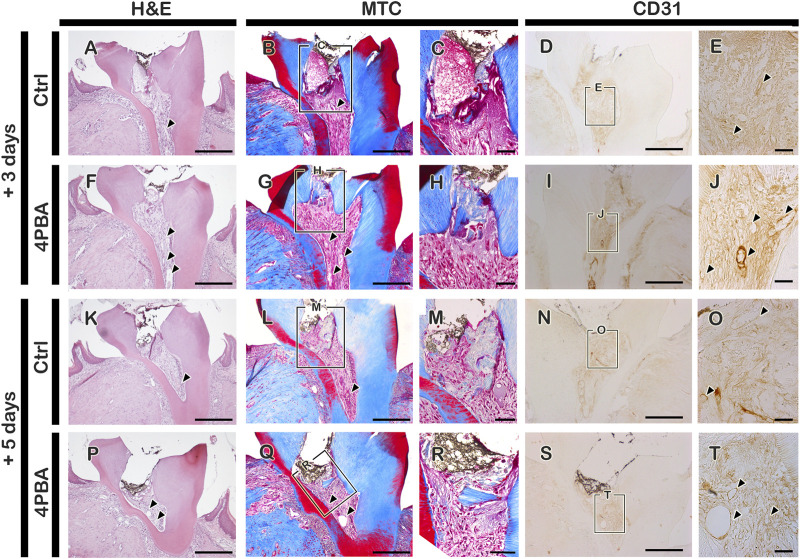
Histological examination using H&E and MTC staining, and immunostaining of CD31 after pulp access preparation. Arrowhead indicates blood vessels (A-B, E, F-G, J, K-L, O, P-Q, T). Boxes in B, D, G, I, L, N, Q, S indicate enlarged view. *Scale bars*: A-B, D, F-G, I, K-L, N, P-Q, S: 200 μm; C, E, H, J, M, O, R, T: 50 μm.

The mice were sacrificed after 3, 5, and 42 days from local drug treatment to examine the histological and immunostaining analysis. The exposed pulp was sealed with Dycal for protective barrier of applied drugs (4PBA) in the dentin. For immunostainings, mice sacrificed after 3- and 5-days of drug treatment were used. Firstly, we examined the immunostainings of ER-stress related molecules: GRP78 (a key indicator for induction of ER stress), and HRD1 (a marker of ER protein quality control by ER-associated degradation of mis-folded proteins) (Figures 1A–E, H-L, O-S, V-Z). In the 4PBA-treated group, there was a decrease localization patterns of GRP78 in both 3- and 5-days specimens compared to controls (Figures 1A,B, H-I, O-P, V-W). Basal localization pattern of GRP78 was examined to understand the altered localization of GRP78 after drug treatment (Supplementary Figure S1). Interestingly, 4PBA-treated specimens showed similar or less intensity of GRP78 localization when compared with control exposed pulp cavity (Supplementary Figure S1, Figure 1). On the other hand, the localization pattern of HRD1 showed differential expression levels in 3- and 5-days specimens. After 3 days, the control group showed broad cytoplasmic localization pattern of HRD1 at the entire pulp area (Figures 1C–E), whereas 4PBA-treated group showed nucleic localization pattern of HRD1 specifically in the damaged upper pulp cavity (Figure 1J-L). After 5 days, the entire region of pulp cavity in the both control- and 4PBA-treated groups showed nucleic localization of HRD1 (Fig. 1Q–S, X-Z). Furthermore, we also examined MPO localization to evaluate the early modulation pattern of inflammation in the exposed pulp (Figures 1F,G, M-N, T-U, A′-B′). After 3 days, there was not much differences in the intensity of immunostaining, however, after 5 days, the control group showed stronger positive reaction of MPO when compared with 4PBA-treated group (Figures 1F,G, M-N, T-U, A′-B′). Histological changes in the pulp and dentin of the upper molar were evaluated using H&E and Masson’s trichrome staining (Figures 2A–C, F-H, K-M, P-R). After 3- and 5-days of drug treatment, compared to controls, 4PBA-treated specimens showed more obvious blood vessel formation in the pulp and adjacent dentin wall (Figure 2A-T, arrow heads) and more number of cells developed nuclear heterochromatin staining patterns (Figures 2A–C, F-H, K-M, P-R). It was further confirmed by the immunostaining against endothelial cell marker (CD31), which showed more number of CD31 immunopositive cells in the 4PBA-treated group when compared to control (Figures 2D,E,I,J, N-O S-T). In addition, beneath the exposed pulp area, increased cell number which showed plasma-like structure were detected in the 4PBA-treated group (Figures 2H,R). This histogenesis and the altered localization patterns of proteins, including GRP78, HRD1, MPO and CD31 suggest that 4PBA treatment would modulate inflammation reaction and resolve ER stress along with facilitation of blood vessel formation in the early stage of pulp exposure. Furthermore, the intensities of immunostainings were quantified as none (-), exist (+), strong (++) and strongest (+++) and prepared as a supplementary table 1.

### Micro-CT and Dentin Bridge Evaluations

**FIGURE 3:**
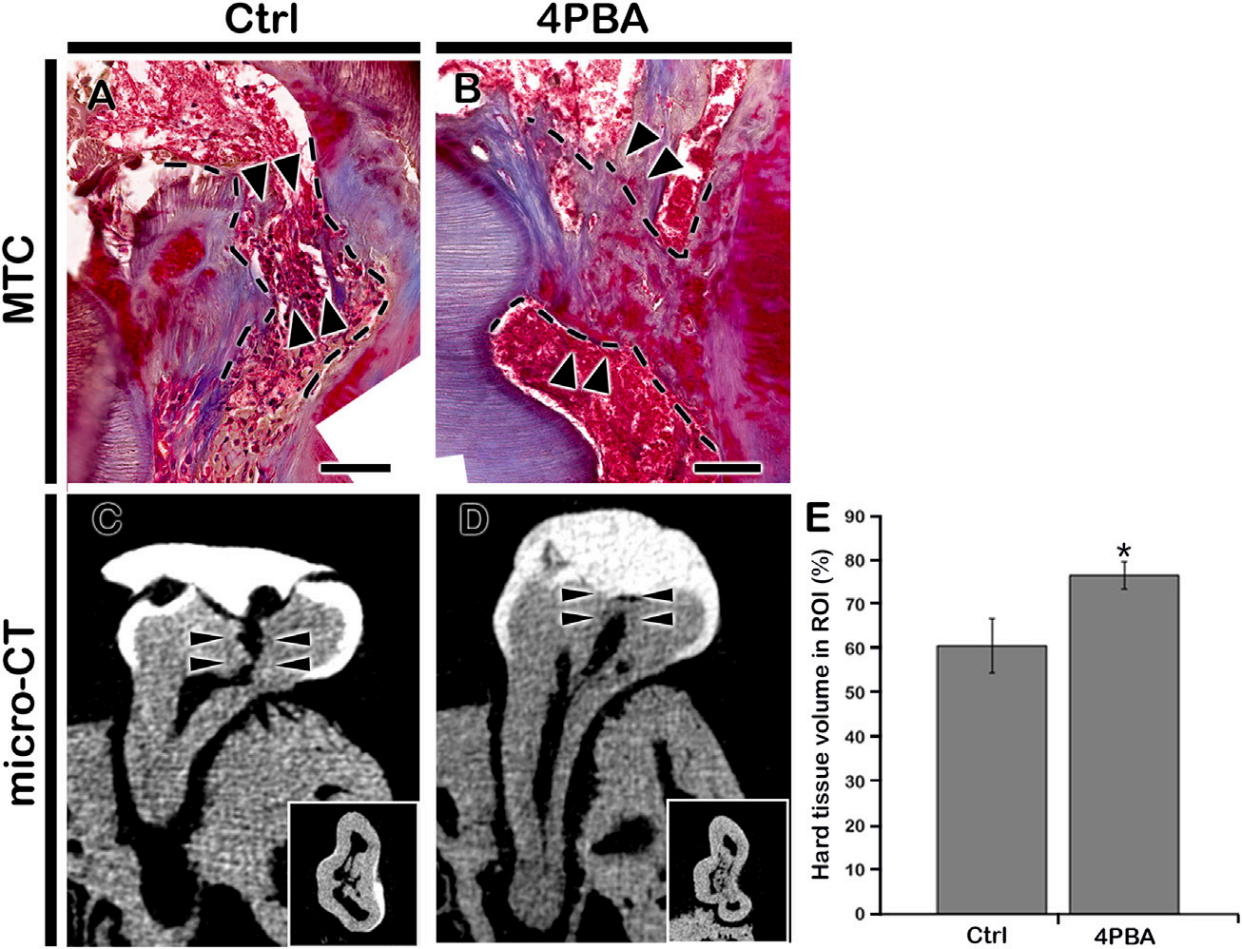
Micro-CT examinations and MTC staining after 42 days from cavity preparation. MTC staining showing dentin-bridge (arrows) formation in the 4PBA- treated specimen **(A–B)**. Micro-CT showing pulpal access preparation and dentin-bridge formation after 42 days of cavity preparation **(C–D)**. The percentage of hard tissue formation within the region of interest (N = 3) **(E)**. ROI, region of interest. Insets in C and D indicate occlusal view. Arrowheads indicate region of interest and dotted lines indicate formation of dentin bridge. * indicates *p < 0.05* **(E)**. Scale bars: 50 μm: A-B.

After 42 days of 4PBA treatment, MTC staining and micro-CT evaluation were employed to examine the dentin-bridge and percentage of newly regenerated tissue in the pulp cavity. In the control group, the hard tissue deposition appeared to be incomplete, and some inflammatory cells were still observed below the exposed areas (Figure 3A). In contrast, the 4PBA-treated group showed complete dentin bridge formation of which dentinal tubules are absent and irregularly arranged, similar with a bone-like structure; regenerated hard tissue continues with reparative dentin (Figure 3B). Similarly, the micro-CT of coronal and occlusal views of 42 days after cavity preparation showed significant differences in the quality of the regenerated hard tissue (Figures 3C–E). The hard tissue density of the 4PBA group was higher than that of the control group (Figures 3C–E). The percentage of hard tissue in the region of interest marked with arrowheads in the control group was 60.7 ± 6.2%; N = 3, whereas it was 76.7 ± 3.1%; N = 3 in the 4PBA-treated group (Figure 3E).

### Altered Immunostainings of NESTIN and TGF-β1

**FIGURE 4:**
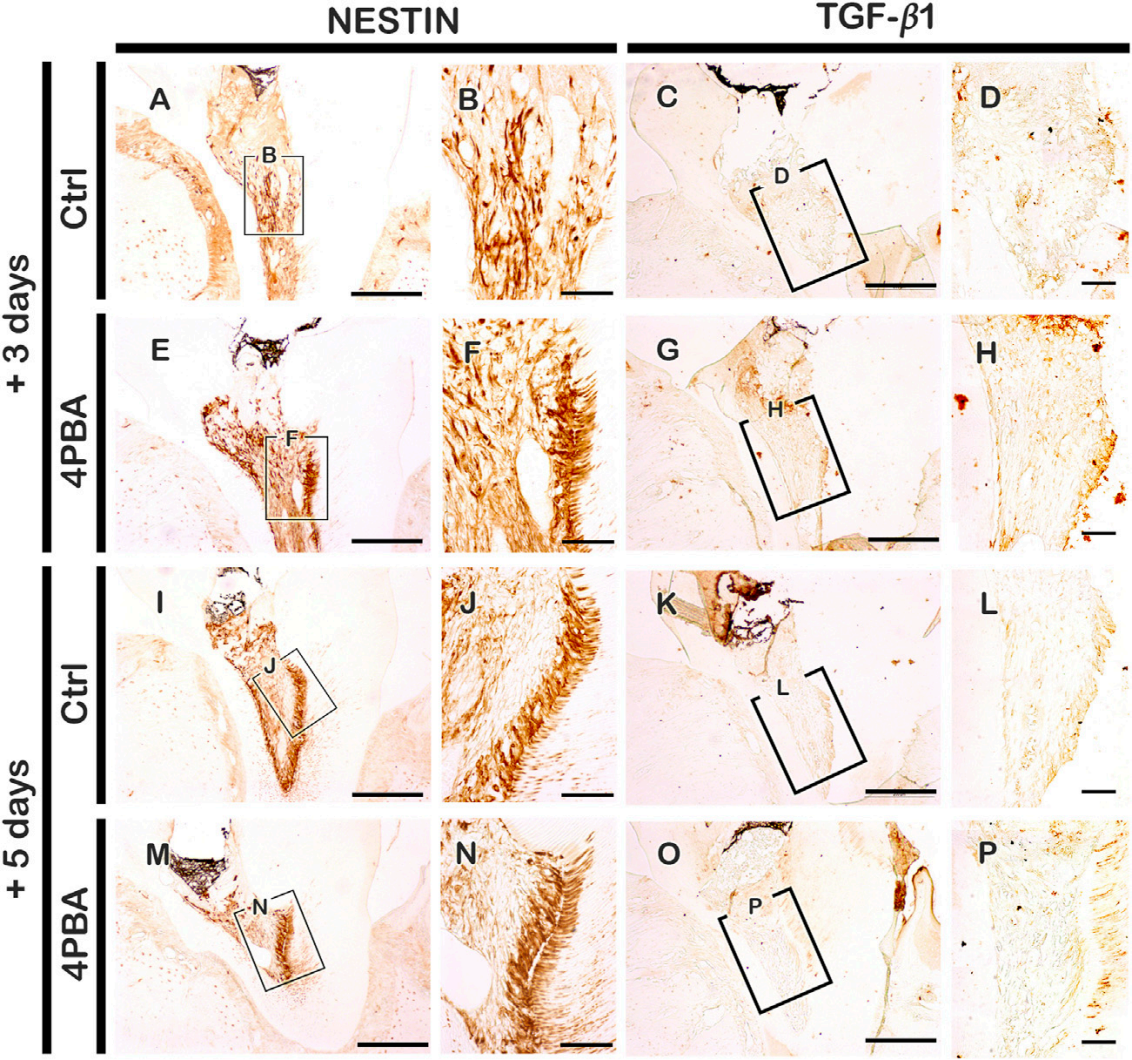
Immunohistochemistry staining with NESTIN and TGF-β1 after 3 and 5 days pulp access preparation. Boxes in A, C, E, G, J, K, M, O indicate enlarged view in B, F, J, N, D, H, L, P. *Scale bars*: A, C, E, G, I, K, M, O: 200 μm; B, F, J, N, D, H, L, P: 50 μm.

According to the histological changes between the 4PBA and control groups, an immunolocalization pattern for NESTIN was performed at 3 and 5 days after cavity preparation (Figures 4A,B, E-F, I-J, M-N). After 3 days, NESTIN, an indicator of active odontoblasts, was more strongly localized in cells along the dentin wall in the 4PBA-treated group (Figure 4F) than it did in the control group after 3 days (Figure 4B). Similarly, after 5 days from 4PBA treatment, the localization of NESTIN showed stronger positive reactions in active odontoblasts than those of control (Figures 4J,N). In addition, localization of TGF-β1, known as an important regulator of various cellular events including proliferation, differentiation, and reparative dentinogenesis, was also carefully examined (Figures 4C,D, G-H, K-L, O-P). After 3 days, the 4PBA-treated group showed stronger positive reaction against TGF-β1 at the exposed pulp area and dentin wall (Figure 4H) when compared to controls (Figure 4D), however, after 5 days, the control- and 4PBA-treated groups showed almost similar localization patterns of TGF-β1 in the dental pulp (Fig. 4L, P). These localization patterns of TGF-β1 would suggest that 4PBA treatment activates the earlier TGF-β1 expression in the exposed pulp cavity.

## Discussion

Formation of dentin is mainly contributed by cellular physiology of odontoblasts. These specific secreting cells are precisely regulated and modulated by ER regulation and vesicle trafficking to form the specifically featured dentin matrices through protein synthesis, folding, trafficking, secretion, and patterned apposition (Brookes et al., 2014; Aryal et al., 2019; Liang et al., 2019). Previous studies reported that modulation of ER stress would be an important cellular pathway for differentiation and maintenance of dentin (Kim et al., 2014; Brookes et al., 2017). In this study, we hypothesized that in the exposed dentin and pulp complex, ER stress would be occurred unavoidably to repair the dentin structure and maintain the vitality of the pulp as examined in other wound sites (Brookes et al., 2014; Aryal et al., 2019; Liang et al., 2019). In inflammation condition, a number of studies revealed the vicious circle of escalation of pathological conditions between ER stress and inflammation responses (Luo et al., 2010; Qi et al., 2011). Inflammation disturbs protein folding, leading to the accumulation of unfolded or mis-folded proteins inside the ER which in turn results in UPR (Kim et al., 2013). Furthermore, sustained ER stress induces chronic activation of UPR, which would eventually lead to severe inflammation and even cell death (Rao et al., 2001). Recently, studies on ER regulation and vesicle trafficking made use of chemical chaperone treatments to subdue a range of incurable diseases (Singh et al., 2008; Luo et al., 2010; Wu et al., 2020). Similarly, in this study, in order to modulate the excessive inflammatory environment and promote dentin regeneration after pulp exposure, we examined the function of the chemical chaperone 4PBA, one of the candidate drugs for relieving ER stress, an important cellular step for protein formation and secretion in regeneration, and as a drug repositioning approach, in exposed pulp cavity for 3, 5, and 42 days, using a well-established animal model system (Jung et al., 2017; Aryal et al., 2021).

Recent reports elaborated 4PBA has chaperone properties and stabilizes protein conformation in ER which facilitates ER stress and UPR activation (Ozcan et al., 2006; Ozcan et al., 2009). We performed local delivery of 4PBA with concentration of 100 μM which was determined in *vitro* cell culture condition (Ranga Rao et al., 2018) and it is comparably lower than oral medication for systematic applications (Singh et al., 2008; Basseri et al., 2009; Luo et al., 2010; Brookes et al., 2014). This low concentration of drug local delivery was available in pulp cavity preparation model system and would confirm the merits including harmless, economic and efficient method for treatment. Interestingly, our results showed that local delivery of 4PBA presented the excellent morphological changes with dentin bridge and altered cellular changes in late and early time period of exposed pulp and dentin. Based on results, we proposed that two key factors for successive pulp therapy would be management of ER stress and control of initial inflammatory status of the pulp which influences the quality of newly mineralized hard tissue after treatment. As we expected, 4PBA showed the excellent function for resolving ER stress from the exposed pulp (Figure 1). After precise examinations of localization patterns of ER stress related molecules, GRP78 and HRD1, we suggest that the regulation of inflammation of dental pulp and formation of reparative dentin would be resulted from the proper modulation of ER stress by treatment of 4PBA (Kaufman, 2002; Lee, 2005). HRD1 plays a critical role in ER-associated degradation (ERAD) of mis-folded/unfolded proteins as it protects cells from ER stress-induced cell death (Sun et al., 2015; Wei et al., 2018; Wu et al., 2020). We have observed interesting result after precise examination of localization patterns of HRD1. The differential localization patterns of HRD1 between controls and the 4PBA group is worth noting. The localization pattern of HRD1 in control group showed cytoplasmic positive localization generally at the entire pulp area. In the 4PBA-treated group after 3 days, there was a decrease in the localization compared to controls, and localization pattern was observed only at a limited area of the dentin wall, which was a nuclear localization (Figure 1). These results coincide with a previous report that found that cytoplasmic localization of HRD1 would take place when secretory cells are impaired (Wu et al., 2020). The results support that 4PBA treatment in the exposed dentin alleviates ER stress and ERAD by providing the proper condition of tissue regeneration. In addition, histological observation and the localization pattern of MPO would suggest that 4PBA initiates blood vessel formation for rapid control of inflammation through neutrophil-mediated modulation of inflammation after the pulp exposure.

Moreover, the increased localization patterns of NESTIN and TGF-β1 after 3 days of pulp exposure in our study confirmed the previous report that elevated levels of NESTIN would stimulate cell proliferation and invasion by stimulating the TGF-β1 signaling pathway (Toyono et al., 1997; Bokhari et al., 2016; Niwa et al., 2018). To understand the 4PBA molecular reactions, we examined the micro-CT and histomorphology after 6 weeks of cavity preparation (Figure 3). As we observed at early-onset cellular changes, 4PBA-treated group showed long-term regeneration of reparative dentin in the exposed pulp with dentin bridge formation (Figure 3). Our results showed that 4PBA treatment in exposed pulp and dentin complex would modulate blood vessel formation and also promotes the active odontoblasts to produce reparative dentin through TGF-β1 signaling pathways. We suggest and recommend that 4PBA would be a feasible treatment after pulpal cavity preparation to facilitate dentin regeneration and alleviating ER stress.

## Conclusion

Overall, we examined the function of the FDA-approved drug, 4PBA in dentin regeneration as a drug repositioning approach. This attempt would be a plausible answer to extend and develop new drugs and techniques in the dental field. For example, as a drug repositioning strategy, many ER stress-related drugs such as melatonin, simvastatin, and TUDCA should be tested in the dental field (Ozcan et al., 2009; Ranga Rao et al., 2018; Karakida et al., 2019). In addition, we suggest examining the synergistic effects of ER stress-relieving molecules in combination with previously established treatment including triple antibiotic paste and Wnt signaling-related small molecules (Vijayaraghavan et al., 2012; Jung et al., 2017; Faria et al., 2018).

## Data Availability

The original contributions presented in the study are included in the article/Supplementary Material, further inquiries can be directed to the corresponding authors.
