# Supplementary material for: Facilitation of Reparative Dentin Using a Drug Repositioning Approach With 4-Phenylbutric Acid
Source: Front Physiol. 2022 May 4;13:885593. doi: 10.3389/fphys.2022.885593 (PMC9114641; doi:10.3389/fphys.2022.885593)
Supplement: Supplementary file 1 [file DataSheet1.PDF]

Facilitation of reparative dentin using a drug repositioning approach with 4-Phenylbutric acid

Eui-Seon Lee[1#], Yam Prasad Aryal[1#], Tae-Young Kim[1], Ji-Youn Kim[2], Hitoshi Yamamoto[3], Chang-Hyeon An[4], Seo-Young An[4], Youngkyun Lee[1], Wern-Joo Sohn[5], Jae-Kwang Jung[6], Jung-Hong Ha[7]*, Jae-Young Kim[1]*

[1]Department of Biochemistry, [4]Department of Oral and Maxillofacial Radiology, [6]Department of Oral Medicine, [7]Department of Conservative Dentistry, School of Dentistry, IHBR, Kyungpook National University, Daegu, Korea

[2]Department of Dental Hygiene, Gachon University, Incheon, Korea

[3]Department of Histology and Developmental Biology, Tokyo Dental College, Tokyo, Japan

[5]Pre-Major of Cosmetics and Pharmaceutics, Daegu Haany University, Gyeongsan, Korea

*Corresponding address:

Jae-Young Kim, 2177 Dalgubeol-daero, Joong-gu, Daegu 41940, Korea

Tel: +82-53-420-4998; E-mail: jykim91@knu.ac.kr

ORCID: 0000-0002-6752-5683

Jung-Hong Ha, 2177 Dalgubeol-daero, Joong-gu, Daegu 41940, Korea

Tel: +82-53-600-7624; E-mail: endoking@knu.ac.kr

ORCID: 0000-0002-0469-4324

Supplementary Table 1. Statistical evaluation of immunohistochemical staining against CD31, NESTIN, TGF-β1, GRP78, MPO and HRD1

	Group	CD31	NESTIN	TGF-β1	GRP78	MPO	HRD1	
							Nucleus	Cytosol
+ 3 days	Ctrl	+	+	+	+	+	+	+++
	4PBA	+++	+++	++	-	+	++	++
+ 5 days	Ctrl	+	++	-	+++	+++	++	++
	4PBA	+++	+++	-	++	+	+++	+

-:none, +: exist, ++: strong, +++: strongest

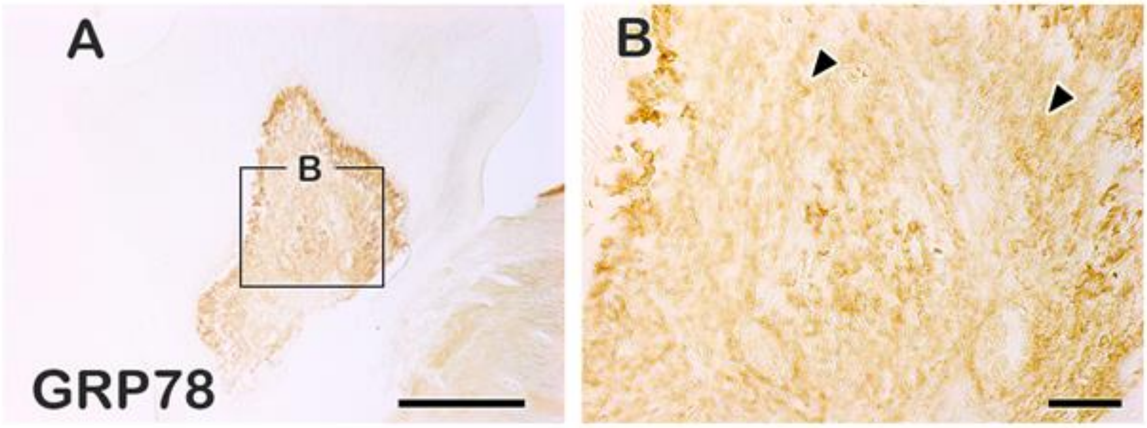

Figure S1: Localization of GRP78 in the non-injured healthy pulp cavity (A-B). Arrowheads indicate GRP78 positive cells. Scale bars: A (200 µm); B (50 µm).
